# Recombinant Expression and Stapling of a Novel Long-Acting GLP-1R Peptide Agonist

**DOI:** 10.3390/molecules25112508

**Published:** 2020-05-28

**Authors:** Sam Lear, Hyosuk Seo, Candy Lee, Lei Lei, Zaid Amso, David Huang, Huafei Zou, Zhihong Zhou, Vân T. B. Nguyen-Tran, Weijun Shen

**Affiliations:** The Scripps Research Institute, d/b/a Calibr, a division of Scripps Research, 11119 North Torrey Pines Road, Suite 100, La Jolla, CA 92037, USA; samlear@scripps.edu (S.L.); hyseo@scripps.edu (H.S.); candylee@scripps.edu (C.L.); leilei@scripps.edu (L.L.); zaidamso@scripps.edu (Z.A.); dhuang@scripps.edu (D.H.); zouhuafei@gmail.com (H.Z.); zhouzhihong0818@yahoo.com.sg (Z.Z.); vnguyent@scripps.edu (V.T.B.N.-T.)

**Keywords:** diabetes, obesity, stapled peptide, recombinant expression, lipidation, semisynthesis, long-acting, PEG, GLP-1R agonist, exenatide

## Abstract

Owing to their pleiotropic metabolic benefits, glucagon-like peptide-1 receptor (GLP-1R) agonists have been successfully utilized for treating metabolic diseases, such as type 2 diabetes and obesity. As part of our efforts in developing long-acting peptide therapeutics, we have previously reported a peptide engineering strategy that combines peptide side chain stapling with covalent integration of a serum protein-binding motif in a single step. Herein, we have used this strategy to develop a second generation extendin-4 analog rigidified with a symmetrical staple, which exhibits an excellent in vivo efficacy in an animal model of diabetes and obesity. To simplify the scale-up manufacturing of the lead GLP-1R agonist, a semisynthesis protocol was successfully developed, which involves recombinant expression of the linear peptide followed by attachment of a polyethylene glycol (PEG)-fatty acid staple in a subsequent chemical reaction step.

## 1. Introduction

Glucagon-like peptide-1 receptor (GLP-1R) agonists have attained blockbuster status as therapeutics in the area of metabolic diseases. In preclinical and clinical studies, GLP-1R agonists have been shown to lower fasting plasma glucose and promote glycemic control through glucose-dependent insulinotropic and glucagonostatic effects, without conferring risks of hypoglycemia [1,2]. The incretin effect resulting from targeting GLP-1R led to the successful commercialization of three short-acting agonists [exenatide, lixisenatide, and liraglutide] and three long-acting agonists [bydureon, dulaglutide, and semaglutide] as therapeutics for type 2 diabetes (T2D) [3]. In addition to the above metabolic outcomes, GLP-1R agonists are able to induce sustained weight loss through appetite suppression and delayed gastric emptying, ultimately leading to the commercialization of the once daily liraglutide for chronic weight management in patients with obesity [4]. More recently, GLP-1R agonists have also shown promise in the clinical studies for management of nonalcoholic steatohepatitis, a liver disease with no approved therapies to date [5,6].

As part of our efforts in developing long-acting therapies for chronic diseases, we have previously utilized the in-house developed polyethylene glycol (PEG)-fatty acid stapling technology to produce ”E6”, a potent, long-acting GLP-1R agonist exhibiting excellent efficacy in animal models of diabetes and obesity [7]. Despite the superior pharmacokinetic properties, the first-generation staples used to synthesize E6 were found to produce mixtures of regioisomers during the stapling reaction. While this did not affect the activity of the resulting conjugates, it was undesirable as chemical heterogeneity presents challenges during manufacturing and pharmacological and toxicological characterizing of the isomeric products. Herein, we report the use of an improved, structurally symmetric staple, which circumvents the above problems by producing only one product upon stapling. Moreover, the methionine residue present in E6 was replaced with leucine to mitigate instability due to oxidation. The lead candidate demonstrates similarly long half-life and excellent in vivo efficacy in controlling blood glucose in mice. In addition, we report the semisynthesis of the lead GLP-1R agonist involving expression of the linear peptide functionalized with chemically reactive cysteine residues, followed by attachment of a PEG-fatty acid staple in a subsequent chemical reaction step. This protocol can be used to prepare large quantity of the target GLP-1R agonist with high purity for clinical testing and subsequent commercialization as a therapeutic for T2D and obesity.

## 2. Results and Discussion

**Figure 1:**
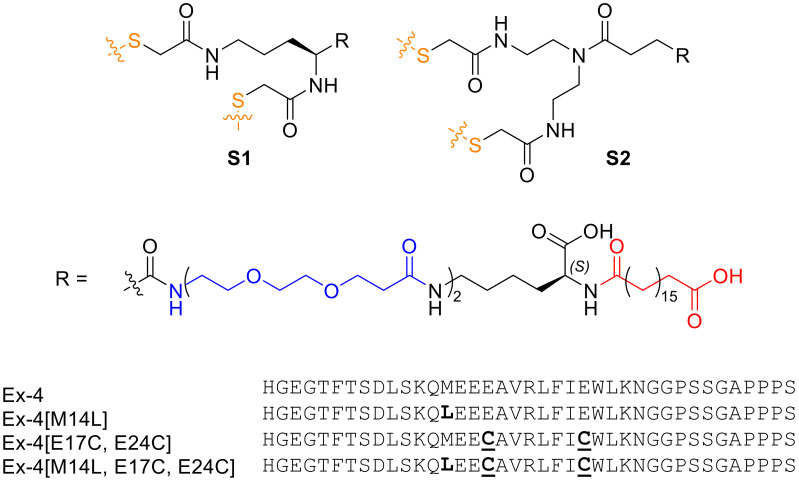
Structures of lipidated moieties, **S1** and **S2**, used for stapling, and exenatide (Ex-4) analog sequences.

Lipidation is an established approach for half-life extension of therapeutic peptides, and has found prominence in the metabolic disease space, where many lipidated peptides and proteins such as GLP-1R agonists (semaglutide [8], liraglutide [9]) and insulin analogs (insulin detemir [10] and degludec [11]) have been approved as therapies for diabetes and obesity. Furthermore, peptide stapling is a technique that can be used to improve the potency and serum stability of bioactive peptides, which shows promise in clinical peptide drug development [12,13]. Previously, we developed a long-acting exenatide (Ex-4) analog stapled at engineered Cys positions within the sequence, incorporating an optimized lipid moiety for enhanced serum protein binding [3,7]. The peptide, Ex-4[E17C, E24C]-**S1** (‘’E6’’), demonstrated significantly improved half-life and in vivo efficacy in chronic obesity models, including enhanced glucose control and body weight reduction. Despite being a promising lead compound, we sought to address the structural asymmetry of the **S1** staple (Figure 1), which renders it susceptible to the formation of regioisomers upon stapling of the diCys-containing precursor peptide. This required the development of a symmetric analog of the staple (**S2**, Figure 1), which cannot form regioisomers and which we have used recently to generate long-acting oxyntomodulin analogs [14].

### 2.1. In Vitro Activity and Pharmacokinetics of Symmetric Staple Analog

**Figure 2:**
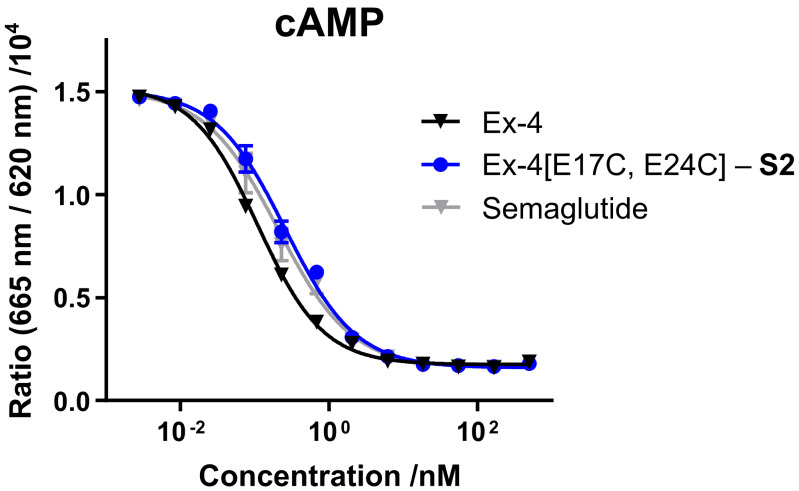
Dose-response curves for glucagon-like peptide-1 receptor (GLP-1R) agonists in intracellular cyclic adenosine monophosphate (cAMP) assay (EC_50_ values shown in Table 1).

Synthesis of staple **S2** and the Ex-4[E17C, E24C] sequence, and stapling of the synthetic peptide were carried out as previously reported [3,14]. In vitro receptor activity of synthetic Ex-4[E17C, E24C]-**S2** was evaluated in mammalian cell lines overexpressing GLP-1R, either via direct readout of intracellular cyclic adenosine monophosphate (cAMP) release, or through a cAMP response element luciferase reporter (CRE luc), and compared with commercial GLP-1R agonist semaglutide (Figure 2 and Table 1). Both **S1**- and **S2**-stapled analogs exhibit similar activity to the native Ex-4 control and semaglutide.

**Figure 3:**
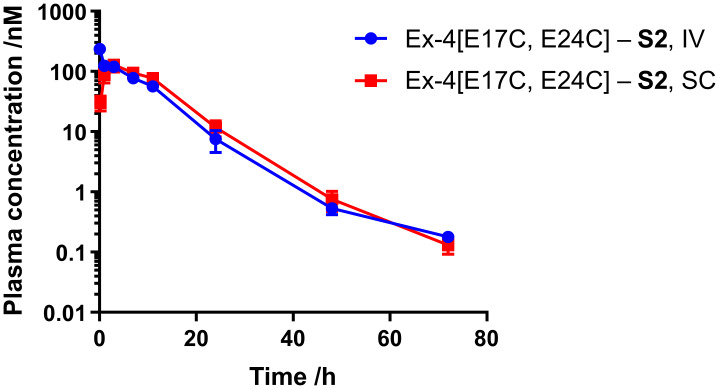
Mouse pharmacokinetic profiles for Ex-4[E17C, E24C]-**S2**. Peptides were administered via intravenous (IV) or subcutaneous (SC) injection to male CD-1 mice (n = 4) at 0.12 mg/kg. Blood samples were collected between 0.25 and 72 h after dosing, and plasma concentration of the peptides was analyzed via CRE luc reporter functional assay, as detailed in the Materials and Methods section.

Next, we sought to assess the half-life of Ex-4[E17C, E24C]-**S2** in mouse. Male CD-1 mice (n = 4) were administered peptide via either SC or IV route at 0.12 mg/kg, and plasma samples were collected at 0.25, 1, 3, 7, 24, 48, and 72 h for SC and 5 min and 1, 3, 7, 24, 48, and 72 h for IV dosing. Peptide plasma concentrations were determined via the CRE luciferase reporter assay, as described. Pharmacokinetic data are given in Figure 3 and Table 2. Ex-4[E17C, E24C]-**S2** exhibits a half-life of over 7 and 9 h via SC and IV administration, respectively, which is comparable to that previously reported for the **S1** analog and semaglutide (data not shown) [7].

### 2.2. In Vivo Efficacy of Symmetric Staple Analog

**Figure 4:**
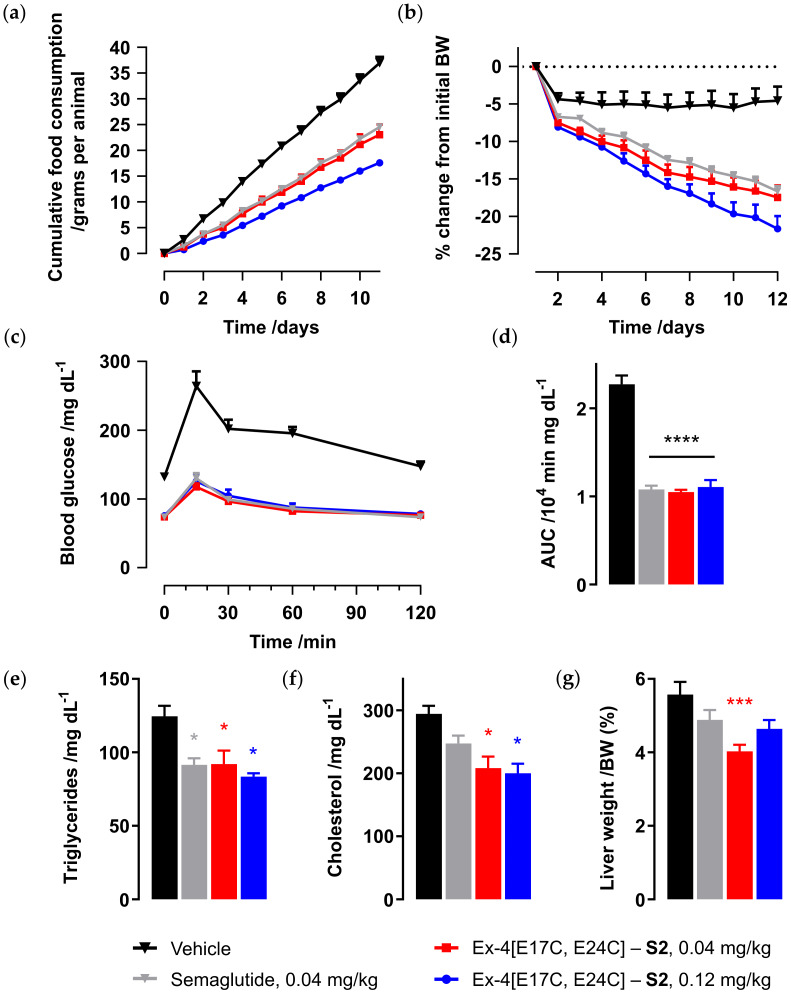
Daily SC dosing of peptide or vehicle control (phosphate-buffered saline) in diet-induced obesity (DIO) mouse model over 12 days (n = 6 per group), first dose administered on day 1 following overnight fast: (**a**) cumulative food intake; (**b**) % change from initial body weight (BW); (**c**) oral glucose tolerance test (OGTT) at day 1 with (**d**) area under the curve (AUC); (**e**) serum triglycerides, (**f**) total cholesterol, and (**g**) liver weight as a percentage of body weight (BW) at day 13; * *p* < 0.05, *** *p* < 0.001, and **** *p* < 0.0001, relative to vehicle control.

Given the confirmed in vitro activity and mouse half-life demonstrated for symmetric staple analog Ex-4[E17C, E24C]-**S2**, the next step was to fully validate efficacy of the new lead peptide in a chronic in vivo diet-induced obesity (DIO) model. Peptide was administered daily to diet-induced obese mice for 12 days, at either 0.04 or 0.12 mg/kg via SC injection, alongside semaglutide (0.04 mg/kg) and vehicle control (Figure 4). Ex-4[E17C, E24C]-**S2** exhibited dose-dependent reduction in food intake (Figure 4a) and body weight (Figure 4b), and comparable efficacy to semaglutide at 0.04 mg/kg in food intake and body weight reduction, glucose control in the oral glucose tolerance test (OGTT) at day 1 (Figure 4c,d), and reduction of serum triglycerides (Figure 4e). Furthermore, our peptide showed a superior reduction of total cholesterol (Figure 4f) and liver weight (Figure 4g), despite the relatively short treatment period.

### 2.3. Recombinant Expression and Stapling of M14L Variant

**Figure 5:**
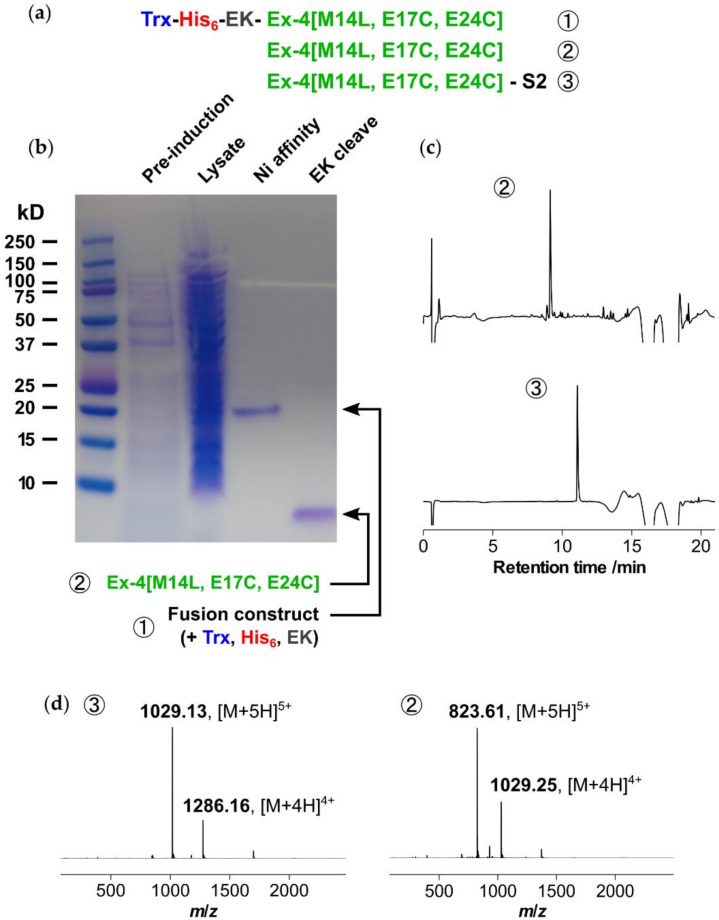
Expression in *E. coli*, characterization and stapling of Ex-4[M14L, E17C, E24C] with **S2**: (**a**) fusion construct for expression, incorporating thioredoxin (Trx) expression tag, His_6_ tag for purification and enterokinase (EK) cleavage site; (**b**) sodium dodecyl sulfate-polyacrylamide gel electrophoresis (SDS-PAGE) showing supernatant following cell lysis, material after Ni affinity purification and subsequent EK cleavage; (**c**) UV chromatogram (absorbance at 214 nm) of recombinantly expressed peptide ② (86% purity), and peptide following stapling and reversed-phase HPLC (RP-HPLC) purification, ③ (96% purity); (**d**) electrospray ionization mass spectrometry (ESI-MS) characterization of ② and ③ (calculated *m*/*z*: ② 823.81 and ③ 1028.93 [M + 5H]^5+^, ② 1029.51, and ③ 1285.92 [M + 4H]^4+^).

Next, a robust method for expression of the linear diCys-functionalized Ex-4[E17C, E24C] peptide needed to be developed, as an alternative to chemical synthesis. In addition, we sought to address the metabolic liability associated with the presence of a methionine side chain, which is sensitive to oxidation. This was solved by a simple Met-to-Leu substitution, which has previously been validated for exenatide as resulting in no loss of potency against the GLP-1 receptor [15,16]. We anticipated that soluble expression would be best suited for the relatively short and highly soluble Ex-4[M14L, E17C, E24C] sequence, and to this end a thioredoxin (Trx) expression system was selected, as shown in Figure 5 [17,18]. A His_6_ tag was incorporated to enable purification via immobilized metal affinity chromatography (IMAC) using nickel sepharose resin, with an enterokinase (EK) cleavage site for subsequent removal of the N-terminal tags.

The expression construct was cloned into a pET28a vector and expressed in *E. coli* as the cytoplasmic, soluble fraction. After culturing overnight at 37 °C using kanamycin selection, expression was induced at OD_600_ = 0.8 and the culture was incubated for 4 h. Following centrifugation, lysis of the cell pellet, and further centrifugation, the supernatant containing soluble fusion was purified using nickel affinity chromatography with elution at 200 mM imidazole to yield ~30 mg/L of protein. Selective cleavage of the Trx and His_6_ tags was achieved via enterokinase digestion overnight at room temperature. After concentration the cleaved product, Ex-4[M14L, E17C, E24C], was purified via reversed-phase HPLC (RP-HPLC) to yield 4 mg/L of peptide. The purified linear peptide was then stapled as previously reported, using dibromoacetyl-**S2** in acetonitrile/ammonium bicarbonate buffer (pH 8.5) for 4 h, followed by acidification with acetic acid and purification via a second RP-HPLC step.

### 2.4. Activity, Pharmacokinetics, and in Vivo Efficacy of Semisynthetic M14L Analog

**Figure 6:**
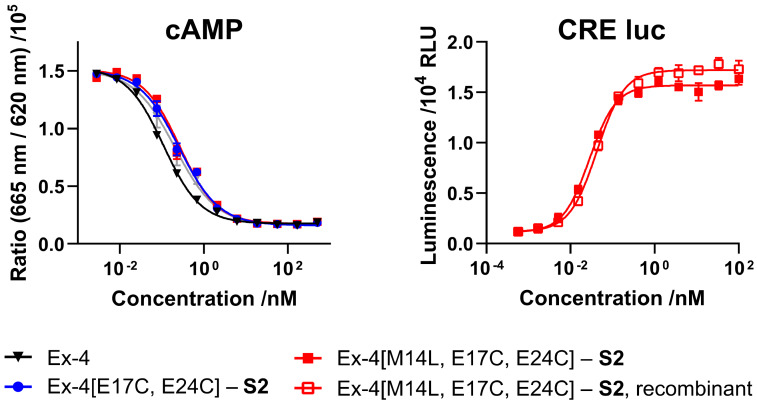
Dose-response curves for chemically synthesized and semisynthetic GLP-1R agonists in intracellular cAMP assay or CRE luciferase reporter assay (see Materials and Methods section, EC_50_ values shown in Table 3).

Activity of semisynthetic Ex-4[M14L, E17C, E24C]-**S2** was evaluated using cAMP and CRE luc reporter assays (Figure 6 and Table 3). As expected, the Met-to-Leu analog demonstrates equal or better potency than the native methionine-containing peptide, and the semisynthetic (recombinant) product exhibits comparable activity to the fully synthetic material.

**Figure 7:**
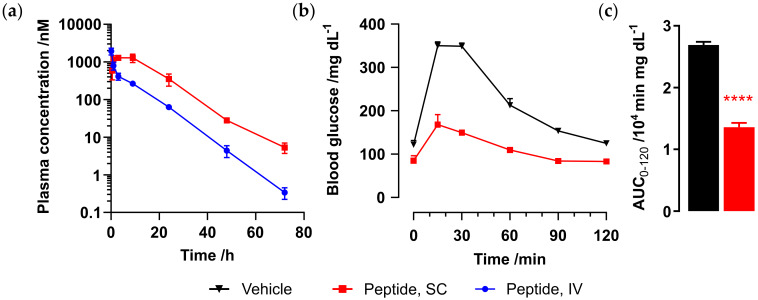
(**a**) Mouse pharmacokinetic data for Ex-4[M14L, E17C, E24C]-**S2** administered via intravenous (IV) or subcutaneous (SC) injection to male CD-1 mice (n = 3), blood samples were collected between 0.5 and 48 h after dosing, and plasma concentration of the peptides was analyzed via CRE luc reporter functional assay, as detailed in the Materials and Methods section; (**b**) oral glucose tolerance test (OGTT) 1 h after administration of vehicle (phosphate-buffered saline, PBS, pH 8.2, black line) or Ex-4[M14L, E17C, E24C]-**S2** (0.05 mg/kg, SC); **** *p* < 0.0001, relative to vehicle control; (**c**) refers to plasma glucose levels obtained by measuring the area under the curve (AUC).

The half-life of Ex-4[M14L, E17C, E24C]-**S2** was evaluated in mouse, as carried out for the Met analog (Figure 7a). Male CD-1 mice (n = 3) were administered peptide via either SC (1 mg/kg) or IV (0.3 mg/kg) route, and plasma samples were collected at 0.5, 1, 3, 7, 24, 48, and 72 h for SC and 5 min and 1, 3, 7, 24, 48, and 72 h for IV dosing. Peptide plasma concentrations were determined as previously carried out via CRE luc reporter assay. Pharmacokinetic parameters are given in Table 4. Ex-4[M14L, E17C, E24C]-**S2** exhibits an IV half-life of over 6 h and up to 8 h via SC administration, which is comparable to that observed for Ex-4[E17C, E24C]-**S2**.

In order to demonstrate a blood glucose-lowering effect upon administration of Ex-4[M14L, E17C, E24C]-**S2,** we carried out an oral glucose tolerance test (OGTT) in C57BL/6 mice. Mice were challenged with 2 g/kg oral glucose 1 h after dosing of either vehicle (n = 4) or peptide. Significant glucose control was observed upon SC administration of Ex-4[M14L, E17C, E24C]-**S2** (0.05 mg/kg, n = 2), as shown in Figure 7b,c.

The promising data described demonstrate that both the staple modification and Met-to-Leu substitution do not adversely affect the in vivo pharmacokinetics or efficacy of our stapled long-acting Ex-4 peptide, demonstrating the potential for development of the new optimized lead Ex-4[M14L, E17C, E24C]-**S2** as a once-weekly dosed GLP-1R agonist.

## 3. Materials and Methods

Unless otherwise noted, all reagents were purchased from commercial suppliers (MilliporeSigma, Merck KGaA, Darmstadt, Germany; Fisher Scientific, Waltham, MA, USA) and used without further purification. Chemically-synthesized peptides were purchased from Cellmano Biotech Limited (Hefei, China), InnoPep (San Diego, CA, USA), Shanghai Apeptide Co. (Shanghai, China), or Shanghai Dechi Biosciences Co. (Shanghai, China). Staples **S1** and **S2** were purchased from WuXi Apptec Co. (Shanghai, China) and synthesized as previously reported [7,14]. Semipreparative reversed-phase HPLC (RP-HPLC) was performed on an Agilent 1200 HPLC with a Phenomenex Luna column (C18, 100 Å pore size, 5 µm particle size, 150 × 21.2 mm, flow: 20 mL/min) using A (H_2_O, 0.05% TFA) and B (MeCN, 0.05% TFA). High-resolution mass spectra (HRMS) were recorded on an Agilent 1200 Series Accurate Mass Time-of-Flight (TOF) with an Aeris Widepore column (Phenomenex, Torrance, CA, USA) (XB-C8, 3.6 µm particle size, 150 × 2.1 mm, flow: 0.5 mL/min). Solvents: A—H_2_O + 0.1% formic acid, B—MeCN + 0.1% formic acid, gradient: 0–2 min 5% B, 2–12 min 5–60% B, 12–13 min 60–80% B, 13–14 min 80–20% B, 14–15 min 20–80% B, 15–16 min 80–20% B, 16–17 min 20–95% B, 17–20 min 95% B, and 20–21 min 95–5% B. All animal care and experimental procedures were approved by the Institutional Animal Care and Use Committee (IACUC) of the California Institute for Biomedical Research (Calibr) and strictly followed the NIH guidelines for humane treatment of animals.

### 3.1. Recombinant Expression of Ex-4[M14L, E17C, E24C]

Ex4[M14L, E17C, E24C] fused to N-terminal thioredoxin (Trx) and His_6_ tags (MSDKIIHLTDDSFDTDVLKADGAILVDFWAEWCGPCKMIAPILDEIADEYQGKLTVAKLNIDQNPGTAPKYGIRGIPTLLLFKNGEVAATKVGALSKGQLKEFLDANLAGSGSGSMGSSHHHHHHSSGDDDDKHGEGTFTSDLSKQLEECAVRLFICWLKNGGPSSGAPPPS) was successfully cloned into a pET28a vector, with an enterokinase cleavage site (DDDDK) engineered between the His-tag and exenatide sequence. The fusion protein was recombinantly expressed in *E. coli* (BL21 AI) as the cytoplasmic, soluble fraction. For expression, an overnight culture in 10 mL of LB/kanamycin was diluted 1:100 in 1 L of LB/kanamycin and cultured at 37 °C until OD_600_ reached 0.8. 1 mM IPTG and 0.2% arabinose was added to the culture, which was incubated at 37 °C for 4 h and then centrifuged at 5000 rpm for 20 min. The pellet was dissolved in 100 mM Tris (pH 8), 300 mM NaCl, 10 mM imidazole, and 1 mM PMSF, followed by lysis with a homogenizer at 18000 psi, three times. The lysate was then centrifuged at 10,000 rpm for 20 min at 4 °C to clarify the lysed cell suspension and the supernatant was incubated with prewashed Ni-NTA resin in 100 mM Tris (pH 8), 300 mM NaCl, 10 mM imidazole, and 1 mM PMSF, for 1 h at RT. The protein was eluted with 100 mM Tris (pH 8), 300 mM NaCl, and 200 mM imidazole, to yield ~30 mg/L. The Trx tag was selectively cleaved by enterokinase 100 IU/5 mg for 16 h at RT in 20 mM Tris (pH 7.4), 50 mM NaCl, and 2 mM CaCl_2_. After digestion, the solution was concentrated using an Amicon 10K MWCO filter, followed by RP-HPLC purification (30–50% B over 40 min), to yield 4 mg/L of peptide (86% purity by HPLC).

### 3.2. Peptide Stapling

DiCys-containing peptide was dissolved at a concentration of 2 mM with 1.5 eq of bromoacetyl staple in 1:3 (*v/v*) MeCN/30 mM NH_4_HCO_3_ buffer (pH 8.5). The pH of the reaction mixture was readjusted with ammonium hydroxide to correct the drop in pH caused by the peptide TFA counterion. More MeCN was added for particularly insoluble peptides. The reaction was stirred at RT for 2–4 h and monitored by LC-MS, before acidification to pH 5 via dropwise addition of acetic acid. The resulting solution was lyophilized and purified by RP-HPLC (40–60% B over 40 min) (12% yield after purification, 96% final purity by HPLC).

### 3.3. Intracellular cAMP Assay

To measure peptide-induced GLP-1R-mediated stimulation of cyclic adenosine monophosphate (cAMP) production, a cAMP homogenous time resolved fluorescence (HTRF) assay was performed according to the manufacturer’s instructions (cAMP-Gs Dynamic kit, Cisbio). Briefly, cAMP Hunter CHO cells expressing GLP-1R (DiscoveRx) were seeded overnight in white 384-well plates at 5000 cells per well in 20 μL of F12 medium at 37 °C and 5% CO_2_. The following day, the medium was removed and replaced with 20 μL of Opti-MEM (Gibco) without FBS. Peptides (prepared as 5X solution in Opti-MEM) of different concentrations, and forskolin (a direct activator of adenylate cyclase enzyme, final concentration 10 μM), were added and incubated for 30 min at 37 °C. Detection reagent was added and further incubated for 60 min at room temperature, and read on a compatible HTRF reader (PHERAstar). Concentration-response curves were determined by nonlinear regression analysis using the Prism software (GraphPad Software Inc.).

### 3.4. CRE Luciferase Reporter Assay

HEK293 cells were infected with lentivirus encoding firefly luciferase gene under the control of cAMP responsive element (CRE) promoter (Qiagen, Venlo, Netherlands) and then were selected using 1 μg/mL puromycin (Life Technologies, Carlsbad, CA, USA) for 1 week. The surviving cells (referred to as CRE-HEK293) were expanded and then transfected with a G418 selective mammalian expression plasmid encoding human GLP-1R. The GLP-1R plasmid was transfected into CRE-HEK293 cells using Lipofectamine 2000 and selected with 400 μg/mL geneticin (Life Technologies, Carlsbad, CA, USA). A single colony stable cell line overexpressing both CRE-luciferase and GLP-1R (HEK293-GLP-1R-CRE) was then established for the in vitro activity assay. HEK293-GLP-1R-CRE cells were seeded in 384-well plates at a density of 5000 cells per well and cultured for 18 h in DMEM with 10% FBS at 37 °C and 5% CO_2_. Cells were treated with peptides for 24 h, and receptor activation was reported by luminescence intensities, using One-Glo (Promega, WI) luciferase reagent as per the manufacturer’s instructions. The EC_50_ value for each peptide was determined using GraphPad Prism 6 software (GraphPad, San Diego, CA, USA).

### 3.5. In Vivo Pharmacokinetics Studies

All animal care and experimental procedures were approved by the Institutional Animal Care and Use Committee (IACUC, protocol number CR6-002) of the California Institute for Biomedical Research (Calibr) and strictly followed the NIH guidelines for humane treatment of animals. Male CD-1 mice (age 10 weeks, n = 4 per group) were administrated with 100 μL of each peptide in phosphate buffered saline by SC or IV route. Blood (50 µL) was extracted into heparin tubes and centrifuged at 10000 rpm for 5 min. Collection time points were as follows, SC: 0.25 or 0.5, 1, 3, 7, 10, 24, 48, and 72 h; IV: 5 min and 1, 3, 7, 10, 24, 48, and 72 h. The resulting supernatant plasma were then stored at −80 °C. The concentrations of peptides in plasma at each time point were determined by cell-based CRE luciferase reporter assay as described. Peptide concentrations in plasma were obtained and plotted against time points to obtain in vivo half-life of each peptide, using WinNonLin Phoenix software (Pharsight Corp, St. Louis, MO, USA).

### 3.6. Diet-Induced Obesity (DIO) Mouse Body Weight Model

Diet-induced obesity (DIO) model male mice (age 18 weeks from Taconic Biosciences, La Jolla, CA, USA) maintained on high fat diet (D12492, 60% fat diet) for 12 weeks were administered peptide by daily subcutaneous injection for up to 12 days (n = 6, group housed two per cage, regular light cycle). The average body weight at the beginning of the experiment was 50 g. Mouse body weight and food intake was measured daily from day 0 onwards. Mice were fasted overnight prior to the oral glucose tolerance test (OGTT) on day 1, and then dosed with peptide. After 6 h, 1 g of glucose solution per kg body weight was administered orally, and mouse tail blood glucose levels were measured before (0 min) and after glucose challenge for 2 h. The terminal bleed was collected to evaluate metabolic profile (serum triglycerides and total cholesterol). The data were compared using the unpaired Student’s *t*-test. Where appropriate, data were compared using repeated measures or one-way analysis of variance, followed by the Student-Newman-Keuls post-hoc test.

### 3.7. Wild Type in Vivo Oral Glucose Tolerance Test (OGTT)

C57BL/6 male mice (age 10 weeks) were fasted overnight (~16 h) and treated with vehicle (n = 4) or peptide via SC injection (n = 2) 1 h prior to oral glucose tolerance test (OGTT). After 1 h, 2 g of glucose solution per kg body weight was administered orally, and mouse tail blood glucose levels were measured before (T_0_, 0 min) and after glucose challenge for 2 h. The data were compared using the unpaired Student’s *t*-test. Where appropriate, data were compared using repeated measures or one-way analysis of variance, followed by the Student-Newman-Keuls post-hoc test.

## 4. Conclusions

We have demonstrated a robust semisynthetic protocol for the production of a long-acting exenatide analog, Ex-4[M14L, E17C, E24C]-**S2**, involving recombinant expression of the linear precursor peptide followed by stapling using Cys-reactive chemistry. Furthermore, the methionine residue susceptible to oxidation in the native exenatide sequence was replaced by leucine, and the newly developed symmetric staple **S2** was used in order to circumvent regioisomer formation occuring during synthesis of the first-generation long-acting **S1**-stapled exenatide previously reported. All analogs—including the semisynthetic material—retained comparable in vitro GLP-1R activity in cell-based assays. The staple modification was shown to cause no adverse effect on half-life in mouse, and full validation of in vivo efficacy for the **S2** analog was carried out in a diet-induced obesity mouse model. An in vivo half-life of 8 h was established for Ex-4[M14L, E17C, E24C]-**S2**. SC efficacy was also demonstrated for this analog in an in vivo glucose control study. The expression protocol for the long-acting stapled exenatide analogs described, which contain only proteinogenic amino acids in the peptide backbone, will enable low-cost production at scales not possible using chemical synthesis alone. Given the impressive PK and efficacy demonstrated here, these results pave the way for development of Ex-4[M14L, E17C, E24C]-**S2** as a GLP-1R agonist therapeutic.

## 5. Patents

The lead molecules described and their potential application are covered in U.S. Provisional Patent Application No. 62/994,791, which has been filed.

## Figures and Tables

**Table 1:** Activity of chemically-synthesized GLP-1R agonist sequences stapled with **S1** or **S2** (cAMP responsive element (CRE) luciferase reporter assay and intracellular cAMP assay, see Materials and Methods section).

Peptide	Staple	GLP-1R EC_50_ /pM	
		CRE	cAMP
Ex-4	-	37 ± 3	111 ± 6
Ex-4[E17C, E24C]	**S1**	39 ± 3	-
Ex-4[E17C, E24C]	**S2**	-	260 ± 20
Semaglutide	-	24 ± 2	210 ± 20

**Table 2:** Mouse pharmacokinetic data for Ex-4[E17C, E24C]-**S2**.

Parameter		IV	SC
Dose	/mg·kg^−1^	0.12	0.12
T_½_	/h	9 ± 1	7.4 ± 0.4
CL	/mL·min^−1^·kg^−1^	0.24 ± 0.04	0.22 ± 0.05
T_max_	/h	0.08 ± 0	3 ± 0
C_max_	/μg·mL^−1^	1.2 ± 0.2	0.7 ± 0.2
AUC_all_	/h·µg mL^−1^	8 ± 1	9 ± 2
V_z_	/L·kg^−1^	0.20 ± 0.06	0.14 ± 0.04

**Table 3:** Activity of chemically-synthesized and recombinantly expressed (semisynthetic) GLP-1R agonist sequences, with/without M14L mutation, stapled with **S2** (CRE luciferase reporter assay and intracellular cAMP assay, see Materials and Methods section).

Peptide	Origin	Staple	GLP-1R EC_50_ /pM	
			CRE	cAMP
Ex-4	Synthetic	-	37 ± 3	111 ± 6
Ex-4[E17C, E24C]	Synthetic	**S2**	-	260 ± 20
Ex-4[M14L, E17C, E24C]	Synthetic	**S2**	28 ± 2	270 ± 30
Ex-4[M14L, E17C, E24C]	Recombinant	**S2**	43 ± 2	-

**Table 4:** Mouse pharmacokinetic data for Ex-4[M14L, E17C, E24C]-**S2**.

Parameter		IV	SC
Dose	/mg·kg^−1^	0.3	1
T_½_	/h	6.3 ± 0.4	8.0 ± 0.3
CL	/mL·min^−1^·kg^−1^	0.14 ± 0.01	0.14 ± 0.04
T_max_	/h	0.08 ± 0	4 ± 2
C_max_	/μg·mL^−1^	10 ± 2	7 ± 1
AUC_all_	/h·µg·mL^−1^	35 ± 3	120 ± 30
V_ss_	/L·kg^−1^	0.077 ± 0.005	0.10 ± 0.03
